# Breast Contrast-Enhanced Ultrasound: Is a Scoring System Feasible? ----A Preliminary Study in China

**DOI:** 10.1371/journal.pone.0105517

**Published:** 2014-08-18

**Authors:** Xiaoyun Xiao, Bing Ou, Haiyun Yang, Huan Wu, Baoming Luo

**Affiliations:** Department of Ultrasound, Sun Yat-sen Memorial Hospital, Sun Yat-sen University, Guangzhou, China; University of California, San Diego, United States of America

## Abstract

**Objectives:**

Although many studies about breast contrast-enhanced ultrasound had been conducted, clear diagnostic criteria for evaluating enhancement patterns are still lacking. This study aims to identify significant indicators for breast contrast-enhanced ultrasound and to establish an initial scoring system.

**Materials and Methods:**

Totally 839 patients were included in the study. This study was divided into two parts. 364 patients were included in part 1 while 475 in part 2. Conventional ultrasound and contrast-enhanced ultrasound were used to examine each lesion. Only the cases in part 2 were also examined by elastography. In part 1, Logistic regression analysis was performed to predict significant variables. A 5-point scoring system was developed based on the results. In part 2, the scoring system was used to evaluate all the breast lesions. To evaluate the diagnostic efficacy of the new scoring system, it was compared with the system established for elastography and conventional ultrasound (BI-RADS).

**Results:**

Three independent variables, namely, lesion scope, margin, and shape were selected in the final step of the logistic regression analysis in part 1. In part 2, the area under the ROC (receiver operating characteristic) curve for the contrast-enhanced scoring system was 0.912. The difference in the diagnostic capabilities of the contrast-enhanced scoring system and elastography was not statistically significant (*P* = 0.17). The difference in the diagnostic capabilities of the contrast-enhanced scoring system and BI-RADS was statistically significant (*P*<0.001).

**Conclusions:**

The contrast-enhanced patterns of benign and malignant breast tumors are different. The application of a 5-point scoring system for contrast-enhanced ultrasound is clinically promising.

## Introduction

The incidence of breast cancer increases each year, and an increasing number of young women suffer from this disease [1]. How to prevent is unknown. The only method to improve the effectiveness of treatment and reduce the death rate is early detection through screening. The prognosis of breast cancer detected in early phases is good [2]. Most Chinese women have relatively small and dense breasts, complicating the interpretation of traditional mammography images [3]. Therefore, sonography is used as the primary clinical work-up tool for Chinese women.

With the development of new techniques, ultrasound now plays an important part in the diagnosis of breast lesions [4]. New imaging technologies include three-dimensional ultrasound, elastography and contrast-enhanced ultrasound (CEUS). Using these techniques, breast lesions can be analyzed in terms of shape, elasticity, and flow. Two-dimensional (2D) ultrasound is the basis of breast cancer diagnosis. Breast imaging reporting and data system (BI-RADS) has also been used for breast ultrasound and can facilitate treatment selection. A lesion categorized as BI-RADS 4 requires a biopsy or short-term follow-up. The incidence of malignancy in these lesions ranges from 3%–94% [5]. Our goal is to reduce unnecessary biopsies and increase diagnostic accuracy through the use of a single examination. New ultrasonic techniques offer this possibility. The elasticity of breast lesions can be evaluated by elastography. Elastography has been verified as useful in early breast cancer detection [6]–[11], and a diagnostic standard has been developed [11].

CEUS has progressed rapidly in the past two decades [12]–[13]. This technique is based on the detection of blood supply in and around the lesion. In the early 1990s, CEUS was applied for the examination of breast lesions. Most studies involved the enhancement of color Doppler signal using a contrast agent. Tiny vessels of the breast lesions could not be detected by color Doppler, because of the low velocity and patient breathing or heart beat artifacts [14]. Various studies have demonstrated that a contrast agent, which confined to vascular lumen, improved color Doppler signals [15]–[17]. Anatomic and dynamic features were better depicted for differential diagnosis [18]. Benign breast lesion vessels are singular and circumferential, with a regular and tapering course. Malignant breast lesion vessels are tortuous, and vessel knot can be detected [19]. In addition, malignant breast masses have more peripheral vessels than benign breast masses at baseline or after contrast material administration [20]. However, diagnostic capability of contrast-enhanced power Doppler sonography is considered to be limited [21]–[22].

In the past 10 years, breast real-time CEUS has been greatly developed. It involves quantitative and qualitative studies. Quantitative assessment mainly concerns time-intensity curves. After contrast agent injection, bubbles flushed in and out of malignant lesions faster than of benign lesions. Peak enhancement density is higher in malignant lesions [23]–[24]. The good correlation with MRI results has indicated that quantitative assessment is reliable [25]–[27]. Qualitative analysis concerns enhancement patterns that have been reported to be different between benign and malignant lesions [28]–[29]. However, the effectiveness of breast CEUS remains unclear, and the lack of clear diagnostic criteria has limited its wider application.

We attempted to identify significant enhancement patterns for breast tumor differentiation. Binary logistic regression analysis has long been widely used in various areas of medical research. Logistic regression models have several advantages for the multiple variable analysis of etiology, including providing exact probabilities for data that are not normally distributed. So far as we know, this study is the first to analyze breast CEUS pattern using a logistic regression model.

In this study, binary logistic regression was used to analyze the enhancement patterns of breast lesions. A logistic regression model was constructed to identify the most significant indicators. We then attempted to establish an initial diagnosis evaluation system for breast CEUS.

## Patients and Methods

### Patient Population

The study was divided into 2 parts. Part 1, from August 2009 to March 2011, a total of 364 patients (mean age 43, range 12–78) with 382 breast lesions were included in this study. The maximum diameters of the lesions ranged from 3.5 to 43.4 mm; with a mean of (15.4±8.3) mm. Part 2, from April 2011 to June 2013, a total of 475 women (mean age 43 years, range 16–84 years) with 498 breast lesions were included in this study. The maximum diameters of the lesions ranged from 3.0 to 49.0 mm, with a mean of (15.7±8.4) mm. Ultrasonic examinations were performed 1–2 d before surgery or core biopsy. The inclusion criterion was the presence of solid breast lesions on conventional ultrasound. Patients were excluded for any of the following reasons: pregnancy, or breast-feeding, and any previous treatment or interventional diagnosis (BI-RADS VI). All of the 839 patients met the above criteria. The study was approved by the institutional ethics committee of Sun Yat-sen Memorial Hospital, and written informed consent was obtained.

### Ultrasonic Equipment

HV900 (Hitachi Medical, Tokyo, Japan) and iU22 (Philips Medical Systems, Bothell, WA, USA) ultrasonic scanners were used for ultrasonic examinations. CEUS was performed using an iU22 with a 9–3 MHz linear transducer (L9–3) and the contrast agent Sono Vue (Bracco Imaging B.V., Geneva, Switzerland). HV900 was used for elastography.

### Ultrasonic Examination

#### Conventional ultrasound

All examinations were performed by the same sonographer, who had 20 years of experience with breast ultrasound. Bilateral breast ultrasonic scanning was performed to detect possible lesions. Once a breast lesion was detected, the following data were recorded: location, maximum diameter, 2D characteristics, and color Doppler characteristics. The 2D characteristics included shape, margin, inner echo and posterior echo, among others. To improve the detection of slow blood flow in lesions, a low velocity scale and low wall filter were used.

#### Elastography

The probe was placed gently and accurately on the breast surface. The ROI (region of interest) was chosen to cover not only the lesion but also the surrounding tissues. A pressure bar indicating a stable 3∼4 mark represented a satisfactory operation. If the lesion was superficial (located within a depth of 5 mm from the skin), more gel was applied on the skin to increase the distance between the probe and the lesion to obtain more accurate results. All lesions were scored according to the established 5-point scoring system [11]. Only the cases in part 2 were examined by elastography.

#### Contrast-enhanced ultrasound

The plane of a lesion with rich blood or the most irregular shape was chosen as the CEUS target plane. Dual image mode was applied to locate the lesion accurately during the whole procedure. This mode is particularly useful when the lesion is too small to detect. The mechanical index was set at 0.06. The contrast agent was prepared according to the commonly used method. Briefly, 59 µg powder of Sono Vue powder was mixed with 5 ml of saline water followed by shaking to generate the contrast reagent suspension. The contrast agent was administered into the antecubital vein via a 20-gauge cannula. CEUS examination was performed after a bolus injection of 4.8 ml of contrast agent manually via the intravenous cannula, followed by injection of 5–10 ml of saline water. Real-time images were recorded for up to 180 s for further analysis. The selected plane remained unchanged during the examination. The probe was placed gently on the skin to avoid exerting pressure on the lesion, particularly when the lesion was superficial. When evaluating the enhancement patterns of breast lesions, it is recommended to include both the lesion and surrounding tissues in the CEUS image. Therefore, for the lesions with a maximum diameter of more than 40 mm, a 5- to 2-MHz transducer was selected. 5 cases in part 1 and 8 cases in part 2 were examined in this manner. The patients were told to remain still and attempt to maintain eupnea during the examination to minimize motional artifacts.

### Image analysis

All images were read by two sonographers with a minimum of 8 years of experience with breast ultrasound and 2 years of experience with breast CEUS. Both the sonographers were blinded to patients’ clinical data and final pathological results.

#### Part 1

All enhancement patterns of the 382 breast lesions were analyzed by using MVI software equipped in iU22. With this software, subtle changes in each frame could be accurately detected, and enhancement patterns could be observed carefully. A total of 10 features were identified for enhancement patterns: X1, enhanced time compared with surrounding breast tissue (earlier, synchronous or later); X2, enhanced intensity compared with surrounding breast tissue (hyper-enhanced, iso-enhanced, or hypo-enhanced); X3, enhanced direction (centripetal, centrifugal, or diffuse enhancement); X4, internal homogeneity of the lesion (homogeneous or heterogeneous); X5, margin of the lesion after enhancement (clear or not); X6, shape of the lesion (regular or irregular); X7, ring-like enhancement; X8, scope of the lesion (compare the maximal diameter of the lesion in CEUS image with the one in 2D image) [30]; X9, crab claw-like pattern; and X10, perfusion defect. Both doctors provided their opinions. Consensus was reached through discussion if there was any controversy.

#### Part 2

**Figure 1:**
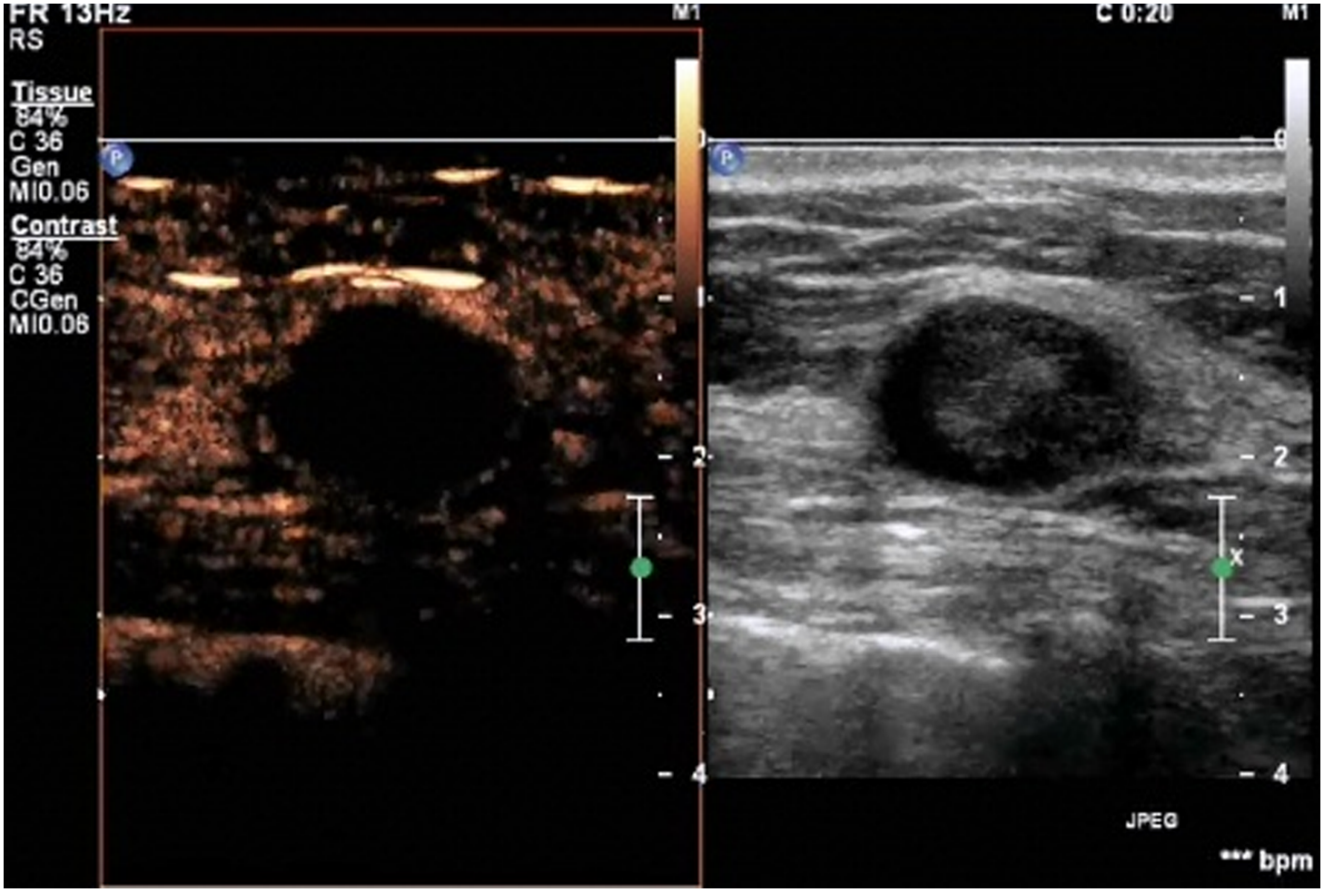
A lesion with a score of 1 by breast contrast-enhanced ultrasound. There is no enhancement in the lesion, with a clear borderline separating the lesion from the surrounding tissue.

**Figure 2:**
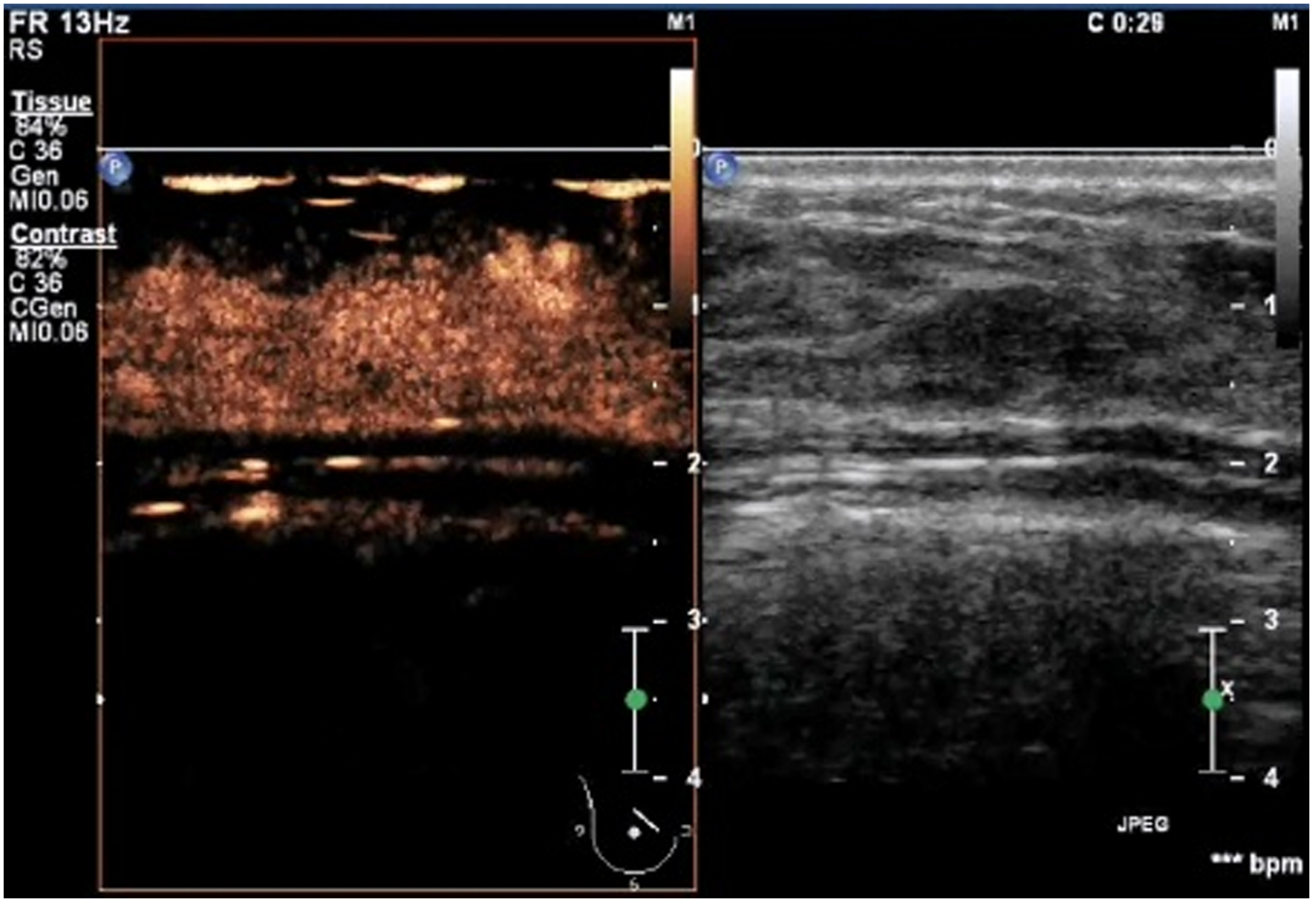
A lesion with a score of 2 by breast contrast-enhanced ultrasound. The lesion displayed iso- and synchronous enhancement with the surrounding tissue, without a clear outline in the contrast-enhanced image.

**Figure 3:**
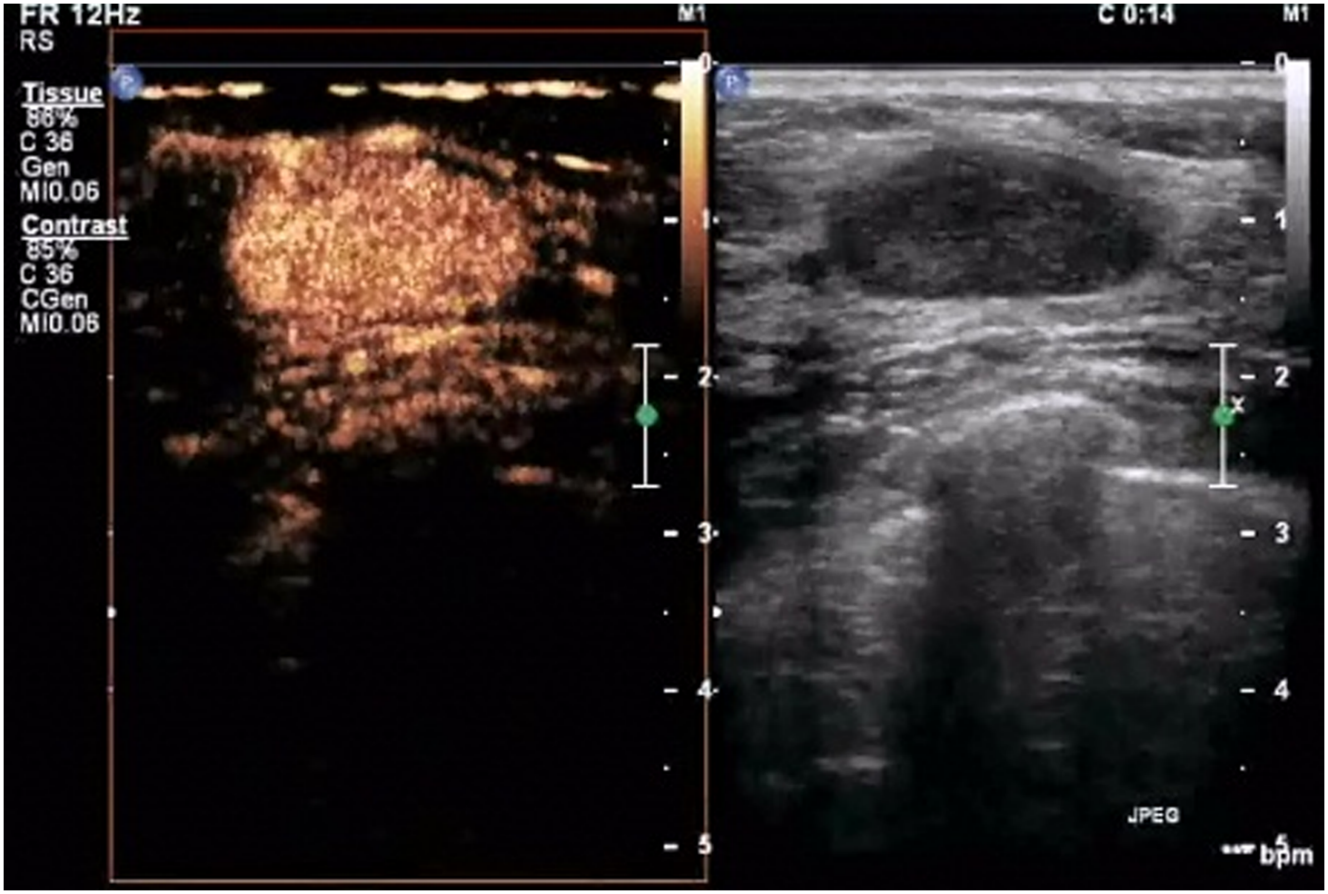
A lesion with a score of 3 by breast contrast-enhanced ultrasound. The lesion exhibits earlier enhancement compared with the surrounding tissue, homogeneous or heterogeneous, with a clear margin (sometimes with ring-like enhancement). The scope of the lesion is almost identical to that shown by 2D ultrasound.

**Figure 4:**
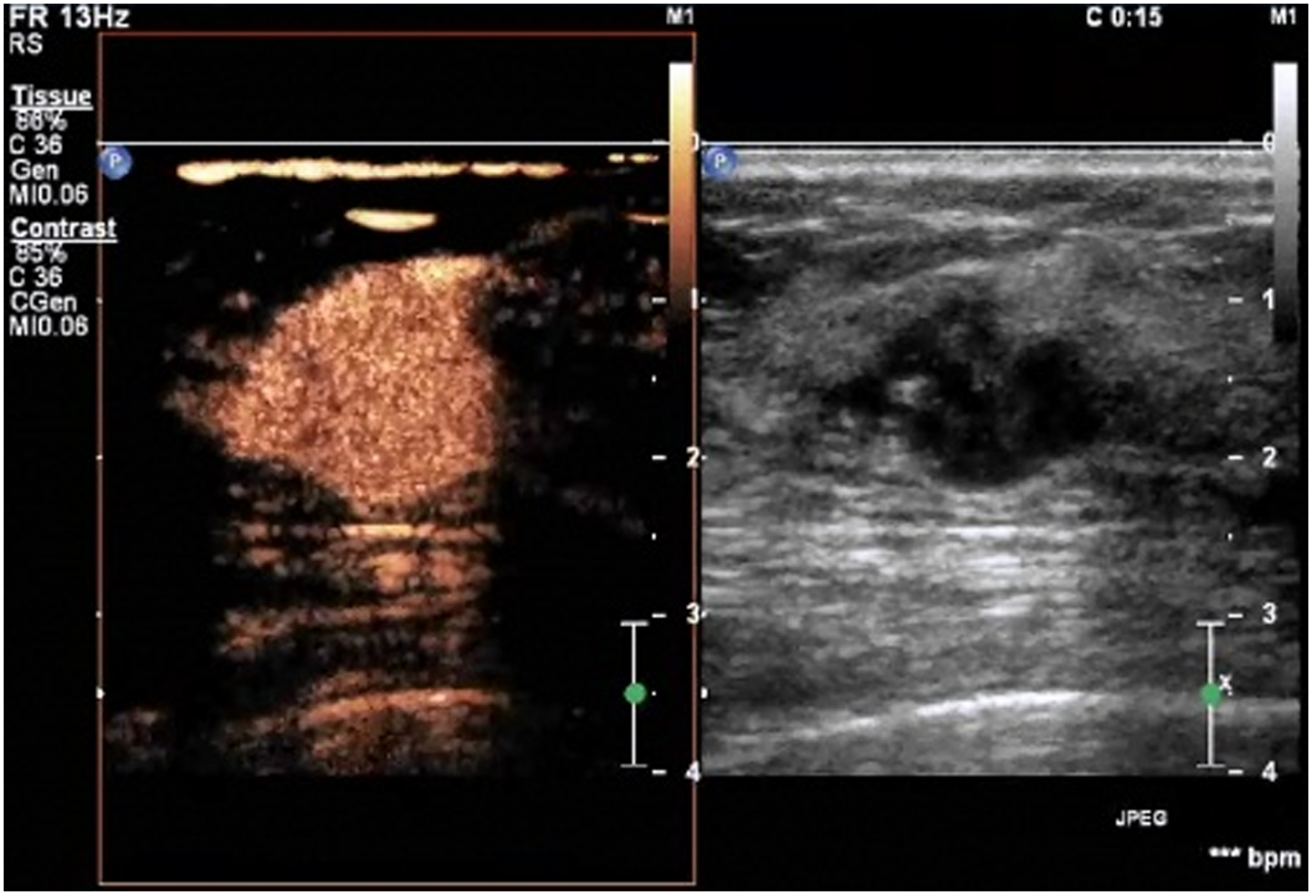
A lesion with a score of 4 by breast contrast-enhanced ultrasound. The lesion displays earlier enhancement than the surrounding tissue, usually heterogeneous. The scope of the lesion in the contrast-enhanced image is larger than in the corresponding 2D image, but the lesion still displays a clear margin, with/without a perfusion defect in the lesions and without crab claw-like enhancement.

**Figure 5:**
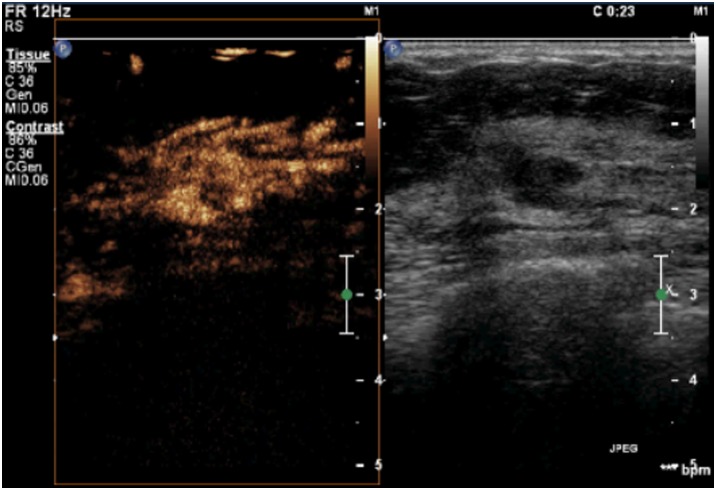
A lesion with a score of 5 by breast contrast-enhanced ultrasound. The lesion is heterogeneously enhanced, with a larger region (compared with that of a 2D image), earlier enhancement, and with/without perfusion defect, particularly with a typical crab claw-like enhancement and an unclear borderline.

We attempted to establish a 5-point scoring system to simplify breast CEUS analysis. The basis of this system is our part 1 study and a literature review. The scoring system is detailed as follow. A score of 1 indicates no enhancement in the lesion, with a clear borderline separating the lesion from the surrounding tissue. A score of 2 indicates that the lesion displayed iso- and synchronous enhancement with the surrounding tissue, without a clear outline in the contrast-enhanced image. A score of 3 indicates that the lesion exhibits earlier enhancement compared with the surrounding tissue, homogeneous or heterogeneous, with a clear margin (sometimes with ring-like enhancement). The scope of the lesion is almost identical to that shown in 2D image. The shape of the lesion is regular: round or oval. A score of 4 indicates that the lesion displays earlier enhancement than the surrounding tissue, usually heterogeneous. The scope of the lesion in the contrast-enhanced image is larger than in the corresponding 2D image, but the lesion still displays a clear margin, with/without a perfusion defect in the lesions and without crab claw-like enhancement. The shape of the lesion is always irregular. A score of 5 indicates that the lesion is heterogeneously enhanced, with a larger scope (compared with that of 2D image), earlier enhancement, and with/without perfusion defect, particularly with a typical crab claw-like enhancement and an unclear margin. The shape of the lesion is always irregular (Figures 1–5). All lesions in this study were scored according to this system.

### Statistical analysis

SPSS 16.0 software (SPSS Inc. Chicago, IL, USA) was used for statistical analysis. For the qualitative analysis in part 1, Chi-square tests were used to examine whether there were significant differences between the enhancement patterns of benign and malignant lesions. Logistic regression was used to identify the useful parameters in the differential diagnosis of breast lesions. For the qualitative analysis in part 2, a ROC curve was constructed to evaluate the diagnostic value of this scoring system. A critical value was generated based on this curve. The diagnostic accuracy, specificity and sensibility were calculated. A Z test was also conducted to compare the AUC between this scoring system and the established elastography scoring system using Medcalc software (version 9.6.4.0). The AUC between this scoring system and BIRADS were also compared. *P*<0.05 was considered significant.

### Pathology analysis

All patients underwent surgery or core biopsy 1–2 days after the ultrasonic examinations. The pathology findings were used as the final diagnostic standard.

## Results

### Pathological results

There were totally 382 lesions in part 1, in which 247 cases were benign and 135 cases were malignant. There were a total of 498 lesions in part 2, of which 291 were benign and 207 were malignant. The results are summarized in Table 1 and 2.

**Table 1:** Final Pathologic Diagnosis of 382 breast lesions in part 1.

Histopathologic Diagnosis	No of lesions
Benign lesions	247
Fibroadenoma	132
Fibrocystic mastopathy	84
Papilloma	13
Chronic Inflammation	8
benign phyllodes tumor	3
Hyperplasia	3
Tubular adenoma	3
Radial scar	1
Malignant lesions	135
Invasive ductal carcinoma	117
Ductal carcinoma in situ	6
Mucinous carcinoma	5
Infiltrating lobular carcinoma	4
Paget disease	2
Solid neuroendocrine carcinoma	1

**Table 2:** Final pathologic diagnosis of 498 breast lesions in part 2.

Histopathologic Diagnosis	No of lesions
Benign lesions	291
Fibroadenoma	153
Fibrocystic mastopathy	89
Intraductal papilloma	16
Chronic mastitis	10
complex sclerosing adenosis	8
benign phyllodes tumor	5
Hyperplasia	2
Tubular adenoma	2
Epidermoid cyst	2
Radial scar	2
Granulomatous mastitis	2
Malignant lesions	207
Invasive ductal carcinoma	158
Ductal carcinoma in situ	27
Mucinous carcinoma	6
Infiltrating lobular carcinoma	6
Invasive papillary carcinoma	3
Paget disease	3
intraductal papillary carcinoma	2
Solid neuroendocrine carcinoma	2

### Qualitative analysis

For part 1 of the study, 10 features of enhancement patterns were observed. Chi-square tests indicated that the differences in the enhancement patterns between malignant and benign lesions were statistically significant (*P* = 0.000). Logistic regression was performed to identify parameters that were important in differentiating breast lesions. Three independent variables were identified in the final step of the logistic regression analysis forward model: scope, margin, and shape.

The model was as follows:

logit (P) = –2.408+2.199×8+1.527×5+1.793×6.

The likelihood ratio test was used to evaluate the fit of the whole model. The fit was significant (χ^2^ = 189.876, *P* = 0.000).

The model was used to predict the malignancy of the 382 breast lesions. When the regression P value is greater than 0.5, the model predicts a malignant tumor and when the regression P value is less than or equal to 0.5, the model predicts a benign tumor. The accuracy was 85.9%.

The probability of a breast lesion being malignant was predicted by the logistic model. A ROC curve was constructed for the predictive value. The area under the ROC curve (Az) was used to evaluate goodness of fit of this model. The Az was 0.852±0.036, with *P*<0.001. These results indicate a good model.

**Figure 6:**
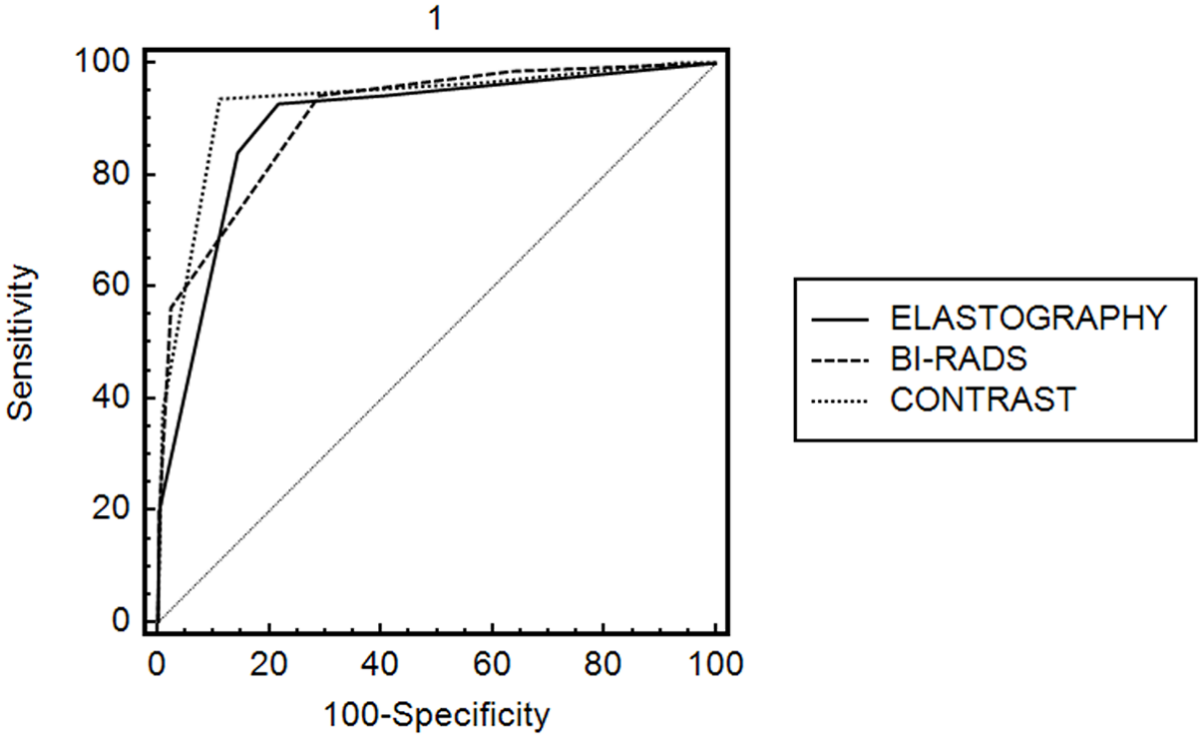
Comparison of ROC curve constructed for breast CEUS, elastography and BI-RADS.

For part 2 of the study, each lesion was evaluated using BIRADS, elastrography, and contrast-enhanced ultrasound (Table 3 and 4). A ROC curve was constructed for the 5-point scoring system of breast CEUS. The AUC was 0.912. The critical value was between 3 and 4. The Youden Index was 0.824. A score of 1–3 represents a benign tumor, whereas a score of 4–5 represents a malignant tumor. The diagnostic accuracy, specificity, and sensitivity of this scoring system were 90.8%, 88.7%, and 93.7%, respectively. The diagnosis accuracy, specificity, and sensitivity of elastography were 87.1%, 88.0%, and 86%, respectively. A ROC curve was constructed for the elastography scoring system. The AUC was 0.892. The difference between the two scoring systems was not significant (Z = 1.374, *P* = 0.17). The diagnosis accuracy, specificity, and sensitivity of BIRADS were 80.7%, 71.1%, and 94.2%, respectively. A ROC curve was constructed for BIRADS. The AUC was 0.827. The difference between the two scoring systems was significant (Z = 3.809, *P*<0.001) (Figure 6).

**Table 3:** Distribution of benign breast lesions by BIRADS, elastography (UE) and contrast-enhanced ultrasound (CEUS) (Totally 291).

BIRADS	Number	UE	Number	CEUS	Number
3	106	1	173	1	20
4A	101	2	55	2	100
4B	76	3	28	3	138
4C	6	4	34	4	31
5	2	5	1	5	2

**Table 4:** Distribution of malignant breast lesions by BIRADS, elastography (UE) and contrast-enhanced ultrasound (CEUS) (Totally 207).

BIRADS	Number	UE	Number	CEUS	Number
3	3	1	3	1	0
4A	9	2	12	2	4
4B	78	3	14	3	9
4C	75	4	136	4	115
5	42	5	42	5	79

## Discussion

CEUS has been applied clinically for years. Its diagnostic accuracy for the differentiation of liver tumors is comparable to that of contrast-enhanced CT/MRI [12]. However, the effectiveness of CEUS in breast lesion diagnosis is still under consideration. So far as we know, there is no clear diagnostic criteria for breast CEUS, which restricts its application. Therefore, we analyzed the enhancement patterns of breast lesions and tried to propose a scoring system.

In part 1, 10 features of the enhancement patterns were observed, and three of these features were identified by the forward logistical model: scope, margin, and shape. This indicates that breast lesions with a larger scope, unclear margin, and irregular shape in contrast-enhanced mode were more likely to be malignant. Previous studies have concluded that gray-scale ultrasound is a reliable method for determining tumor size and is superior to mammography [31]. However, the scope is always underestimated by gray-scale ultrasound compared with pathological specimens [32]–[35]. Most of these underestimate cases involved intraductal carcinoma or diffuse multicenter carcinoma [36]–[38]. Van et al. demonstrated that breast CEUS is a more accurate technique than gray-scale ultrasound for breast lesion size measurement [39]; this conclusion was confirmed by our study. In contrast-enhanced mode, the scope of the malignant lesion was markedly larger than the scope indicated in 2D mode [40]. This phenomenon might be associated with histopathology of malignant lesion. First, 60–70% of the breast cancers were invasive ductal carcinoma, and 85% of the invasive ductal carcinoma composed of carcinoma in situ and invasive carcinoma. Carcinoma in situ is located in the surrounding part of the lesion. If the surrounding part of the lesion in 2D image is without calcification and local ductal dilation, it cannot be detected. In addition, adenosis surrounding malignant lesions may be hypervascular, which increases the scope in contrast-enhanced mode [30]. Although a larger scope was used to indicate malignancy, not all lesions with a larger scope were malignant. Inflammatory lesions also displayed larger scope in contrast-enhanced mode because of the infiltration of inflammatory cells. Unlike benign tumors, malignant tumors were nonencapsulated, with a tendency to infiltrate. Therefore, in contrast-enhanced images, the malignant tumors were irregular, with an unclear margin.

Although not predicted by the logistic regression model, other features were also important for differential diagnosis. For example, a crab claw-like pattern is supposed to be a relatively typical enhancement pattern for malignant tumors [41]. This pattern, which is due to the presence of tortuous vessels, was clearly depicted in contrast-enhanced mode. Malignant lesion cells secrete a variety of angiogenesis factors, particularly VEGF, that promote newborn vessel formation in and, in particular, around the lesions. Vascular endothelial cell around the tumor highly expressed receptor for VEGF [42]–[43]. Histological and electron microscopic studies have indicated that microvessels are located around lesions than in lesions. This may be the pathophysiological basis of the crab claw-like pattern. The presence of radial or penetrating vessels may be one manifestation of tumor invasion [44]. However, claw crab-like enhancement was not required for the diagnosis or exclusion of malignant lesions. Some inflammatory lesions may also display this specific type of enhancement, such as granulomatous mastitis.

A ring-like enhancement pattern was regarded as a typical pattern of benign lesions. Some benign lesions have an intact capsule, which is called a true envelope. In addition, some benign lesions have a false capsule due to the expansion effect of the lesion. In contrast-enhanced mode, the blood supply of the true capsules could be clearly observed as ring-like enhancement pattern. Whereas the lesions with a false capsule only displayed a clear border in contrast images without ring-like enhancement.

A perfusion defect was also an important index for evaluation. Malignant lesions grow faster than benign lesions, and vascular formation and nutrition supply are relatively insufficient. Therefore, part of the tumor may become hypoxic and necrotic [45]. Thus, perfusion defects are often observed in malignant tumors. The vessels of benign lesions are distributed evenly in the lesions. Therefore, necrosis is rarely observed.

Various studies have confirmed that CEUS is advantageous for differentiating breast tumors. However, a systemic evaluation has not been performed. Based on part 1, a scoring system was proposed. All lesions in part 2 were scored according to this system. The ROC curve was generated, and the diagnosis value was set between 3 and 4.

**Figure 7:**
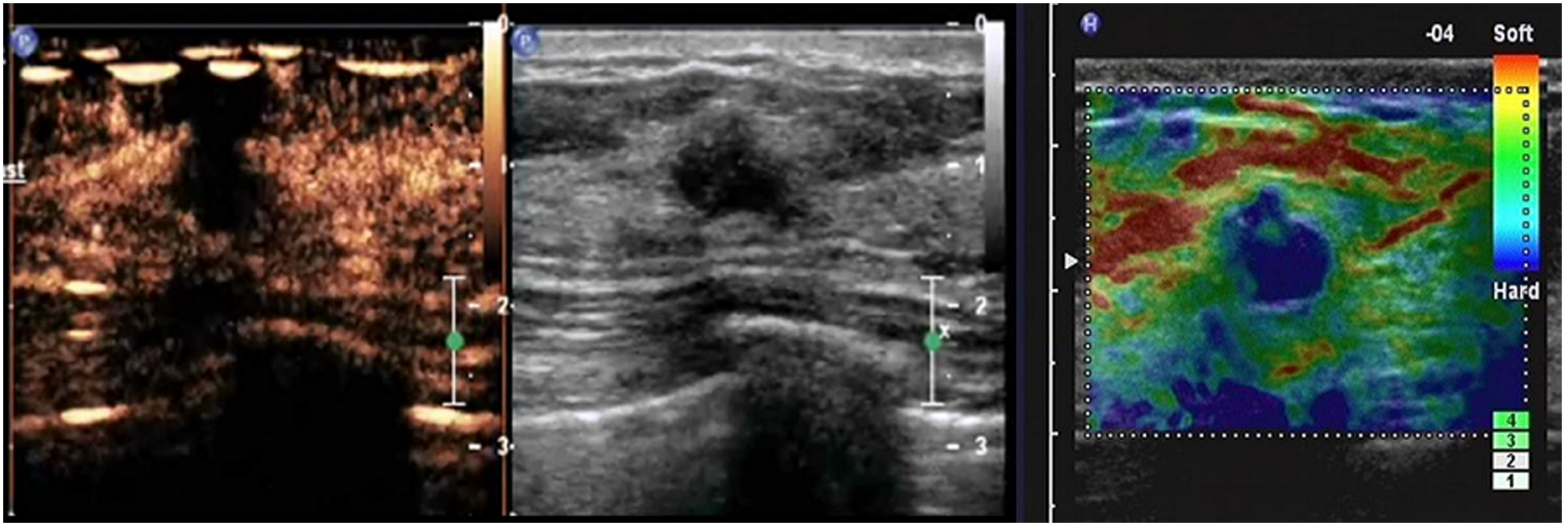
A lesion was categorized as BIRADS 4B, with a score of 4 by elastography, and with a score of 1 by CEUS 5-point scoring system. The pathological result of the lesion was fibrocystic mastopathy with cyst formation.

A total of 20 lesions with a score of 1 were all benign lesions: 2 epidermoid cysts and 18 fibrocystic mastopathies. The lesions all displayed ring-like enhancement without inner enhancement. On sonography, simple cysts are easy to diagnose. It’s classifies as BI-RADS category II based on the assumption that there is less than a 2% probability of malignancy. Complicated cysts are characterized by homogeneous low-level internal echoes and may have a layered appearance. Sometimes, it is difficult to differentiate these cysts from solid lesions. They are categorized as BI-RADS 4, which indicates the need for further intervention. When the lesion is irregular and hard, it mimics a malignant lesion. In this situation, CEUS is useful (Figure 7). When the solid component lacks a blood supply, it could be considered benign. An annual follow-up is adequate, and no further intervention is needed in such a situation [46].

**Figure 8:**
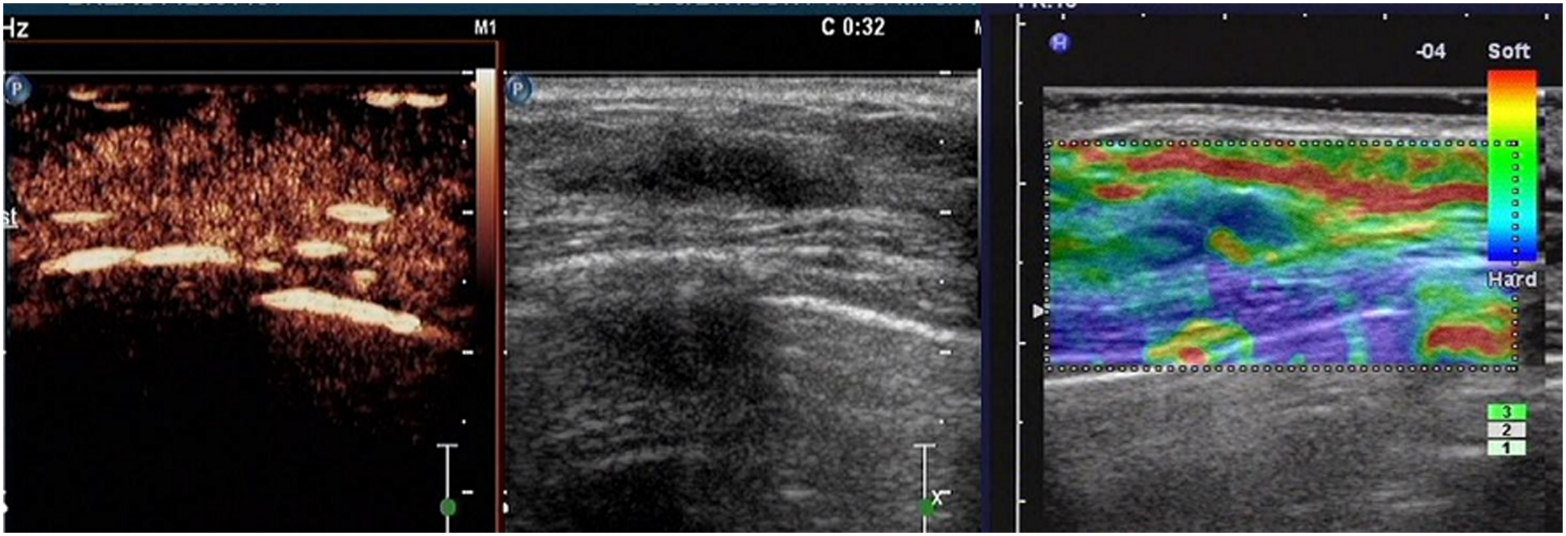
A lesion was categorized as BIRADS 4B, with a score of 4 by elastography, and with a score of 2 by CEUS 5-point scoring system. The pathological result of the lesion was ductal carcinoma in situ.

Of the 104 lesions with a score of 2, 4 were malignant, whereas 100 were benign. A score of 2 indicates that the enhancement pattern of the breast lesion was the same as that of the surrounding breast tissue. In the contrast-enhanced mode, the outline of the lesion could not be delineated. Sono Vue is a true blood agent. The effective vessel diameter from which an echo can be detected is in the capillary range. In our study, we inferred that the blood supply of breast lesions with a score of 2 were the same as that of adjacent breast tissue, and therefore these lesions could not be detected in contrast-enhanced mode. These lesions had the potential to be benign because abnormal vessels were not detected. We correctly diagnosed 100 breast lesions with 4 misdiagnoses. Three were ductal carcinoma in situ and 1 was invasive ductal carcinoma. All three ductal carcinoma in situ lesions were categorized as BI-RADS 4B and displayed an elasticity score of 4. According to their 2D characteristics and elastography, they were correctly diagnosed. They were only mistaken as benign by CEUS (Figure 8). Low-grade carcinoma in situ can depend on normal surrounding vessels for oxygen and nutrition, without eliciting abnormal vessel generation [47]. The lack of malformed neovascularity led to the misdiagnosis. The invasive ductal carcinoma was categorized as BI-RADS 3 with an elasticity score of 3. Not all the lesions demonstrate typical features in imaging. Thus, misdiagnosis is inevitable.

**Figure 9:**
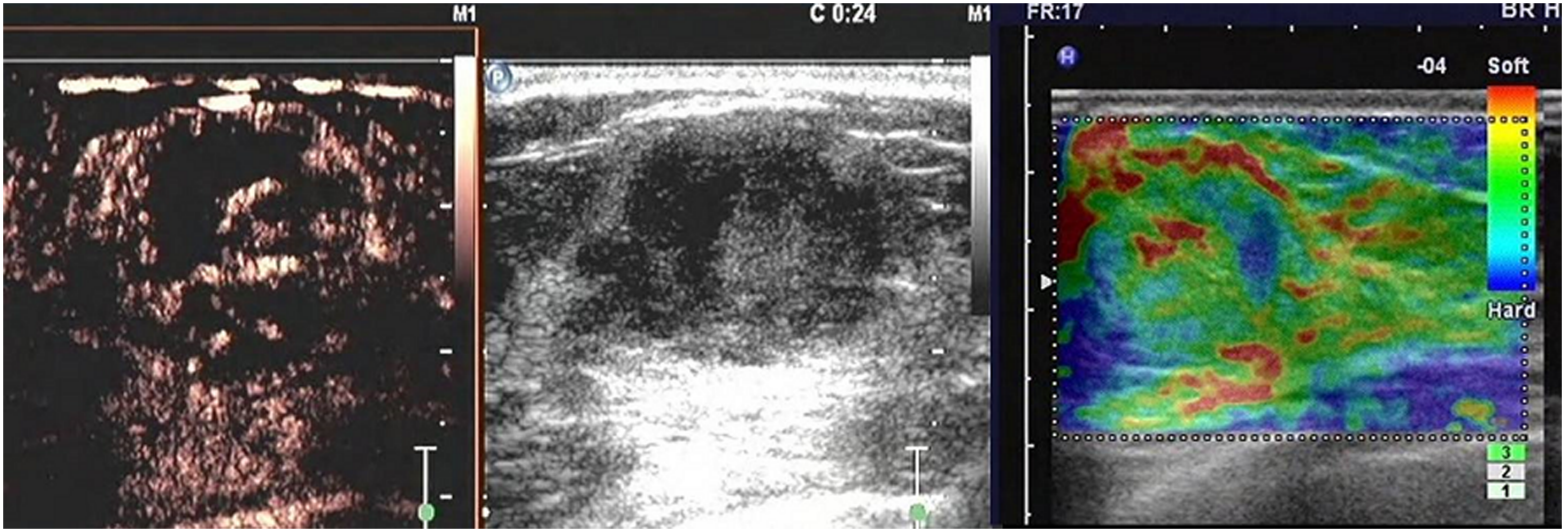
A lesion was categorized as BIRADS 4B, with a score of 1 by elastography, and with a score of 3 by CEUS 5-point scoring system. The pathological result of the lesion was mucinous carcinoma.

Of the 147 breast lesions with a score of 3, 9 were misdiagnosed; eight of these cases were postmenopausal patients. Their breasts were composed of predominately fat rather than glandular tissue. The lesions were located within the fat with little adjacent breast glandular tissue. In contrast-enhanced mode, these lesions were all hyper-enhanced with a clear border and regular shape. No tortuous vessels were observed. Therefore, they were all misdiagnosed as benign. One case was mucinous carcinoma. Pathological findings confirmed the existence of a pseudocapsule that produced a ring-like enhancement pattern, which may have caused the misdiagnosis (Figure 9). However, all 9 lesions were categorized as BI-RADS 4B. Two cases were scored as 1, and 1 case was scored as 3, whereas 6 cases were scored as 4 by elastography.

Once a lesion displayed a larger scope in contrast-enhanced image, it was scored 4 or above. And the lesion was diagnosed as malignant by our algorithm. The presence of the crab claw-like enhancement pattern was the main difference between the score 4 and 5 groups. These results further verified that the crab claw-like pattern is a relatively typical enhancement pattern of malignant lesions.

In total, there were 33 false positive cases in our study. 31 of them scored 4, while 2 scored 5. A total of 9 benign lesions with a score of 4 were mastitis: 7 cases were categorized as BI-RADS 4B, 1 as BI-RADS 4A, and 1 as BI-RADS 4C. By elastography, 6 of the 9 cases were scored as 2, 1 case was scored as 3, and 2 cases were scored as 4. The misdiagnosis might be due to the underestimation of infiltration of inflammatory cells by conventional US. As a result, the scope of mastitis in contrast-enhanced mode was larger than in gray-scale mode. A total of 12 benign cases with a score of 4 were hypervascular fibroadenomas. Intratumoral epithelial hyperplasia is common in fibroadenomas of young women and results in an enhancement pattern overlapping that of a malignant lesion [48]. The other 10 lesions included 5 fibrocystic mastopathies, 4 intraductal papillomas, and 1 cystosarcoma phyllodes (Grade I). We could not determine the reason for the misdiagnosis in these cases. The pathology of 2 benign lesions scored 5 was granulomatous mastitis. Both were categorized as BI-RADS 5 with an elasticity score of 4. This disease is rare. The etiology is unclear, and may be associated with autoimmunity. The lack of experience with the disease is the major reason for the misdiagnosis.

We compared the diagnostic efficacy of this scoring system with that of elastography, which has been previously verified to be useful in the differential diagnosis of breast tumors. The difference in diagnostic efficacy between the two methods was not statistically significant. This encouraging result suggests that this new scoring system will be useful in the future.

As shown in Table 3 and 4, 2D ultrasound had high sensitivity but low specificity, which impaired its overall accuracy. Compared with BI-RADS, the diagnostic specificity was elevated by our scoring system. And the sensitivity was not affected. Breast lesions included in this study were lesions in category 3, 4, and 5 according to the BI-RADS ultrasound lexicon. A total of 46 cases were misdiagnosed by our algorithm in this study. There were two lesions in category 5, one lesion in category 3 and 43 lesions in category 4. Among 43 cases, 12 malignant lesions were missed while 31 benign lesions were over diagnosed. Lesions in category 4 would have an intermediate probability of cancer, ranging from 3 percent to 94 percent. In general, Category 4 lesions require tissue sampling [49]. Previous study reported that heterogeneity, partially indistinct margin, and microlobulation are the most frequent suspicious findings leading to classifying a benign lesion as BI-RADS 4 [50]. If clinicians rely only on 2D ultrasound, excessive biopsies may be performed, consequently eliciting undesirable anxiousness in patients. With contrast-enhanced ultrasound, we hoped to elevate the diagnostic efficacy of breast lesions especially those in category 4. A total of 345 breast lesions in category 4 were included in our research, of which 302 were correctly diagnosed by our algorithm. The diagnostic accuracy was 88.4%. It demonstrated that our algorithm was useful for further differentiation of breast lesions in category 4. However, as we know, the 2D characteristic of untypical benign or malignant breast lesions may mimic each other. So do their blood supply. In our study, we found that enhancement patterns of some breast lesions in category 4 could be very untypical. These lesions were difficult to be correctly diagnosed in the initial research. Another reason for misdiagnosis might be lack of experience. Additional studies are required to analyze untypical enhancement patterns and further improve this scoring system.

### Study Limitations

There were few pathological types of malignant tumors in our study. Most malignant tumors were invasive ductal carcinomas. In our study, we usually chose the plane with rich blood supply or irregular shape for CEUS. The plane remained unchanged during the whole procedure. A single plane may not represent the whole lesion and may result in the loss of important information. Mastitis was misdiagnosed by CEUS. Further exploration is needed to distinguish mastitis from malignant lesions. Not all enhancement patterns were included in the study because the number of cases was limited.

## Conclusions

CEUS is beneficial for breast tumor differential diagnosis. The enhancement patterns of benign and malignant lesions were different. The 5-point scoring system was easy to use and displayed high diagnostic accuracy. Multicenter research is needed to improve this scoring system. It is a promising method for the early diagnosis of breast cancer, which merits further development and evaluation.

## Ethical standards

The clinical research complies with the current laws of China.

## References

[1] Are C, Rajaram S, Are M, Raj H, Anderson BO, et al (2013) A review of global cancer burden: trends, challenges, strategies, and a role for surgeons. J Surg Oncol 107: 221–226. 22926725 10.1002/jso.23248

[2] Suzuki T, Toi M, Saji S, Horiguchi K, Aruga T, et al (2006) Early breast cancer. Int J Clin Oncol 11: 108–119. 16622745 10.1007/s10147-006-0564-7

[3] del Carmen MG, Hughes KS, Halpern E, Rafferty E, Kopans D, et al (2003) Racial differences in mammographic breast density. Cancer 98: 590–596. 12879477 10.1002/cncr.11517

[4] Svensson WE (1997) A review of the current status of breast ultrasound. Eur J Ultrasound 6: 77–101.

[5] Sedgwick E (2011) The breast ultrasound lexicon: breast imaging reporting and data system (BI-RADS). Semin Roentgenol 46: 245–251. 22035666 10.1053/j.ro.2011.04.001

[6] Thomas A, Fischer T, Frey H, Ohlinger R, Grunwald S, et al (2006) Real-time elastography-an advanced method of ultrasound: First results in 108 patients with breast lesions. Ultrasound Obstet Gynecol 28: 335–340. 16909438 10.1002/uog.2823

[7] Zhi H, Ou B, Luo BM, Feng X, Wen YL, et al (2007) Comparison of ultrasound elastography, mammography, and sonography in the diagnosis of solid breast lesions. J Ultrasound Med 26: 807–815. 17526612 10.7863/jum.2007.26.6.807

[8] Cho N, Moon WK, Park JS, Cha JH, Jang M, et al (2008) Nonpalpable breast masses: evaluation by US elastography. Korean J Radiol 9: 111–118. 18385557 10.3348/kjr.2008.9.2.111 PMC2627231

[9] Zhu QL, Jiang YX, Liu JB, Liu H, Sun Q, et al (2008) Real-time ultrasound elastography: its potential role in assessment of breast lesions. Ultrasound Med Biol 34: 1232–1238. 18359145 10.1016/j.ultrasmedbio.2008.01.004

[10] Tan SM, Teh HS, Mancer JF, Poh WT (2008) Improving B mode ultrasound evaluation of breast lesions with real-time ultrasound elastography-a clinical approach. Breast 17: 252–257. 18054231 10.1016/j.breast.2007.10.015

[11] Itoh A, Ueno E, Tohno E, Kamma H, Takahashi H, et al (2006) Breast Disease: clinical application of US elastography for diagnosis. Radiology 239: 341–350. 16484352 10.1148/radiol.2391041676

[12] Xu HX (2009) Contrast-enhanced ultrasound: The evolving applications. World J Radiol 1: 15–24. 21160717 10.4329/wjr.v1.i1.15 PMC2999308

[13] Quaia E (2011) Assessment of tissue perfusion by contrast-enhanced ultrasound. Eur Radiol 21: 604–615. 20927527 10.1007/s00330-010-1965-6

[14] Kettenbach J, Helbich TH, Huber S, Zuna I, Dock W (2005) Computer-assisted quantitative assessment of power Doppler US: effects of microbubble contrast agent in the differentiation of breast tumors. Eur J Radiol 53: 238–244. 15664287 10.1016/j.ejrad.2004.04.017

[15] Schröder RJ, Bostanjoglo M, Hidajat N, Rademaker J, Röttgen R, et al (2002) Analysis of vascularity in breast tumors – comparison of high frequency ultrasound and contrast-enhanced color harmonic imaging. Rofo 174: 1132–1141. 12221572 10.1055/s-2002-33940

[16] Algül A, Balci P, Seçil M, Canda T (2003) Contrast enhanced power Doppler and color Doppler ultrasound in breast masses: Efficiency in diagnosis and contributions to differential diagnosis. Tani Girisim Radyol 9: 199–206. 14661490

[17] Kook SH, Kwag HJ (2003) Value of contrast-enhanced power Doppler sonography using a microbubble echo-enhancing agent in evaluation of small breast lesions. J Clin Ultrasound 31: 227–238. 12767017 10.1002/jcu.10172

[18] Moon WK, Im JG, Noh DY, Han MC (2000) Nonpalpable breast lesions: evaluation with power Doppler US and a microbubble contrast agent–initial experience. Radiology 217: 240–246. 11012451 10.1148/radiology.217.1.r00oc03240

[19] Kedar RP, Cosgrove D, McCready VR, Bamber JC, Carter ER (1996) Microbubble contrast agent for color Doppler US: effect on breast masses. Work in Progress. Radiology 198: 679–686. 8628854 10.1148/radiology.198.3.8628854

[20] Yang WT, Metreweli C, Lam PK, Chang J (2001) Benign and malignant breast masses and axillary nodes: evaluation with echo-enhanced color power Doppler US. Radiology 220: 795–802. 11526284 10.1148/radiol.2203001545

[21] Reinikainen H, Rissanen T, Päivänsalo M, Pääkkö E, Jauhiainen J, et al (2001) B-mode, power Doppler and contrast-enhanced power Doppler ultrasonography in the diagnosis of breast tumors. Acta Radiol 42: 106–113. 11167342

[22] Zdemir A, Kiliç K, Ozdemir H, Yücel C, Andaç S, et al. (2004) Contrast-enhanced power Doppler sonography in breast lesions effect on differential diagnosis after mammography and gray scale sonography. J Ultrasound Med 23: 183–195; quiz 196–197. 10.7863/jum.2004.23.2.183 14992355

[23] Saracco A, Aspelin P, Leifland K, Themudo R, Wilczek B, et al (2009) Bolus compared with continuous infusion of microbubble contrast agent using real-time contrast harmonic imaging ultrasound in breast tumors. Acta Radiol 50: 854–859. 19634024 10.1080/02841850903085576

[24] Balleyguier C, Opolon P, Mathieu MC, Athanasiou A, Garbay JR, et al (2009) New potential and applications of contrast-enhanced ultrasound of the breast: Own investigations and review of the literature. Eur J Radiol 69: 14–23. 18977102 10.1016/j.ejrad.2008.07.037

[25] Caproni N, Marchisio F, Pecchi A, Canossi B, Battista R, et al (2010) Contrast-enhanced ultrasound in the characterization of breast masses: utility of quantitative analysis in comparison with MRI. Eur Radiol 20: 1384–1395. 20033178 10.1007/s00330-009-1690-1

[26] Szabó BK, Aspelin P, Wiberg MK, Boné B (2003) Dynamic MR imaging of the breast. Analysis of kinetic and morphologic diagnostic criteria. Acta Radiol 44: 379–386. 12846687 10.1080/j.1600-0455.2003.00084.x

[27] Ricci P, Cantisani V, Ballesio L, Pagliara E, Sallusti E, et al (2007) Benign and malignant breast lesions: efficacy of real time contrast-enhanced ultrasound vs. magnetic resonance imaging. Ultraschall Med 28: 57–62. 17304413 10.1055/s-2006-927226

[28] Liu H, Jiang YX, Liu JB, Zhu QL, Sun Q (2008) Evaluation of breast lesions with contrast-enhanced ultrasound using the microvascular imaging technique: Initial observations. Breast 17: 532–539. 18534851 10.1016/j.breast.2008.04.004

[29] Zhao H, Xu R, Ouyang Q, Chen L, Dong B, et al (2010) Contrast-enhanced ultrasound is helpful in the differentiation of malignant and benign breast lesions. Eur J Radiol 73: 288–293. 19559551 10.1016/j.ejrad.2009.05.043

[30] Jiang YX, Liu H, Liu JB, Zhu QL, Sun Q, et al (2007) Breast tumor size assessment: comparison of conventional ultrasound and contrast-enhanced ultrasound. Ultrasound Med Biol 33: 1873–1881. 17686569 10.1016/j.ultrasmedbio.2007.06.002

[31] Yang WT, Lam WW, Cheung H, Suen M, King WW, et al (1997) Sonographic, magnetic resonance imaging, and mammographic assessments of preoperative size of breast cancer. J Ultrasound Med 16: 791–797. 9401992 10.7863/jum.1997.16.12.791

[32] Shoma A, Moutamed A, Ameen M, Abdelwahab A (2006) Ultrasound for accurate measurement of invasive breast cancer tumor size. Breast J 12: 252–256. 16684323 10.1111/j.1075-122X.2006.00249.x

[33] Golshan M, Fung BB, Wiley E, Wolfman J, Rademaker A, et al (2004) Prediction of breast cancer size by ultrasound, mammography and core biopsy. Breast 13: 265–271. 15325659 10.1016/j.breast.2004.05.005

[34] Berg WA, Gutierrez L, NessAiver MS, Carter WB, Bhargavan M, et al (2004) Diagnostic accuracy of mammography, clinical examination, US and MR imaging in preoperative assessment of breast cancer. Radiology 233: 830–849. 15486214 10.1148/radiol.2333031484

[35] Heusinger K, Löhberg C, Lux MP, Papadopoulos T, Imhoff K, et al (2005) Assessment of breast cancer tumor size depends on method, histopathology and tumor size itself. Breast Cancer Res Treat 94: 17–23. 16142441 10.1007/s10549-005-6653-x

[36] Hlawatsch A, Teifke A, Schmidt M, Thelen M (2002) Preoperative assessment of breast cancer: Sonography versus MR imaging. Am J Roentgenol 179: 1493–1501. 12438043 10.2214/ajr.179.6.1791493

[37] Berg WA, Gilbreath PL (2000) Multicentric and multifocal cancer: Whole-breast US in preoperative evaluation. Radiology 214: 59–66. 10644102 10.1148/radiology.214.1.r00ja2559

[38] Satake H, Shimamoto K, Sawaki A, Niimi R, Ando Y, et al (2000) Role of ultrasonography in the detection of intraductal spread of breast cancer: correlation with pathologic findings, mammography and MR imaging. Eur Radiol 10: 1726–1732. 11097398 10.1007/s003300000465

[39] van Esser S, Veldhuis WB, van Hillegersberg R, van Diest PJ, Stapper G, et al (2007) Accuracy of contrast-enhanced breast ultrasound for pre-operative tumor size assessment in patients diagnosed with invasive ductal carcinoma of the breast. Cancer Imaging 7: 63–68. 17513187 10.1102/1470-7330.2007.0012 PMC1876179

[40] Zeggelink WF, Deurloo EE, Bartelink H, Rutgers EJ, Gilhuijs KG (2003) Reproducibility of the assessment of tumor extent in the breast using multiple image modalities. Med Phys 30: 2919–2926. 14655939 10.1118/1.1621136

[41] Du J, Li FH, Fang H, Xia JG, Zhu CX (2008) Microvascular architecture of breast lesions: evaluation with contrast-enhanced ultrasonographic micro flow imaging. J Ultrasound Med 27: 833–842; quiz 844. 10.7863/jum.2008.27.6.833 18499843

[42] Brown LF, Berse B, Jackman RW (1995) Expression of vascular permeability factor (vascular endothelial growth factor) and its receptors in breast cancer. Hum Pathol 26: 86–91. 7821921 10.1016/0046-8177(95)90119-1

[43] Lichtenbeld HC, Barendsz-Janson AF, van Essen H, Struijker Boudier H, Griffioen AW, et al (1998) Angiogenic potential of malignant and non-malignant human breast tissues in an in vivo angiogenesis model. Int J Cancer 77: 455–459. 9663610 10.1002/(sici)1097-0215(19980729)77:3<455::aid-ijc23>3.0.co;2-5

[44] Wan CF, Du J, Fang H, Li FH, Zhu JS, et al (2012) Enhancement patterns and parameters of breast cancers at contrast-enhanced US: correlation with prognostic Factors. Radiology 262: 450–459. 22282183 10.1148/radiol.11110789

[45] Metz S, Daldrup-Unk HE, Richter T, Räth C, Ebert W, et al (2003) Detection and quantification of breast tumor necrosis with MR imaging: value of the necrosis-avid contrast agent Gadophrin-3. Acad Radiol 10: 484–490. 12755535 10.1016/s1076-6332(03)80056-9

[46] Berg WA (2005) Sonographically depicted breast clustered microcysts: is follow-up appropriate? Am J Roentgenol 185: 952–959. 16177414 10.2214/AJR.04.0929

[47] Liberman L, Morris EA, Dershaw DD, Abramson AF, Tan LK (2003) Ductal enhancement on MR imaging of the breast. Am J Roentgenol 81: 519–525. 10.2214/ajr.181.2.1810519 12876038

[48] Huber S, Vesely M, Zuna I, Delorme S, Czembirek H (2001) Fibroadenomas: computer-assisted quantitative evaluation of contrast-enhanced power Doppler features and correlation with histopathology. Ultrasound Med Biol 27: 3–11. 11295265 10.1016/s0301-5629(00)00282-9

[49] American College of Radiology. (2003) Breast imaging reporting and data system, 4th ed. Reston, VA: American College of Radiology.

[50] Taskin F, Koseoglu K, Ozbas S, Erkus M, Karaman C (2012) Sonographic features of histopathologically benign solid breast lesions that have been classified as BI-RADS 4 on sonography. J Clin Ultrasound 40: 261–265. 22508447 10.1002/jcu.21923

